# Gamna-Gandy Bodies of the Spleen Detected with Susceptibility Weighted Imaging: Maybe a New Potential Non-Invasive Marker of Esophageal Varices

**DOI:** 10.1371/journal.pone.0055626

**Published:** 2013-01-31

**Authors:** Jiuquan Zhang, Ran Tao, Zhonglan You, Yongming Dai, Yi Fan, Jinguo Cui, Qing Mao, Jian Wang

**Affiliations:** 1 Department of Radiology, Southwest Hospital, Third Military Medical University, Chongqing, China; 2 MR Collaboration NE Asia, Siemens Healthcare, Shanghai, China; 3 Department of Infectious Diseases, Southwest Hospital, Third Military Medical University, Chongqing, China; 4 Department of Radiology, Bethune International Peace Hospital of People's Liberty Army, Shijiazhuang, China; California Pacific Medicial Center Research Institute, United States of America

## Abstract

**Background/Objectives:**

Portal hypertension (PH) is a clinical sequelae of liver cirrhosis, and bleeding from esophageal varices (EV) is a serious complication of PH with significant morbidity and mortality. The aims of this study were to assess the ability of 2D multislice breath-hold susceptibility weighted imaging (SWI) to detect Gamna-Gandy bodies (GGBs) in the spleens of patients with PH and to evaluate the potential role of GGB number as a non-invasive marker of PH and EV.

**Materials and Methods:**

T1-, T2- and T2^*^- weighted imaging and SWI were performed on 135 patients with PH and on 37 control individuals. Platelet counts were collected from all PH patients. Two radiologists analyzed all magnetic resonance imaging (MRI) data, and measured the portal vein diameter, splenic index (SI), and platelet count/spleen diameter ratio. The numbers of patients with GGBs in the spleen were determined, and the numbers of GGB were counted in the four MRI sequences in GGB-positive patients. The portal vein diameter, SI, platelet count, and platelet count/spleen diameter ratio of control individuals were compared with those of GGB-negative and GGB-positive patients on SWI images. The correlations among GGB numbers, the portal vein diameter, the SI, the platelet count, and the platelet count/spleen diameter ratio were analyzed.

**Results:**

The GGB detection rate and the detected GGB number by using SWI were significantly greater than those by using T1-, T2-, and T2*- weighted images. The number of GGBs in the SWI images correlated positively with the portal vein diameter and SI and correlated negatively with the platelet count and platelet count/spleen diameter ratio.

**Conclusion:**

SWI provided more accurate information of GGBs in patients with PH. The number of GGB may be a non-invasive predictor of improving the selection for endoscopic screening of PH patients at risk of EV.

## Introduction

Portal hypertension (PH) is a serious clinical complication of liver fibrosis of different etiologies. The development of PH in cirrhotic liver is related to architectural changes caused by progressive hepatic fibrosis [1]. Congestive splenomegaly is a common finding in patients with PH [2]. Micro-hemorrhages in the splenic parenchyma, caused by a prolonged blood transit time and increased blood pressure secondary to congestive splenomegaly, followed by accumulation of hemosiderin and impregnation of collagen and elastic fibers with iron and calcium were named Gamna-Gandy bodies (GGBs); these are also known as siderotic nodules in the spleen [3], [4].

GGBs can be detected by ultrasonography and computed tomography (CT). Due to the sensitivity to iron-containing structures, GGBs can also be detected with a 9–12% detection rate by magnetic resonance imaging (MRI). GGBs can be demonstrated as multiple tiny foci of decreased signal intensity with all pulse sequences, especially gradient-echo sequences with low flip angle [5]–[8].

Susceptibility-weighted imaging (SWI) exploits the difference in tissues' magnetic susceptibilities to produce a new type of contrast that differs from those obtained with conventional T1- and T2-weighted MR imaging. It is extremely sensitive to changes of susceptibility and enhances the contrast between tissues with different susceptibilities [9]. The applications of SWI in human brain studies have demonstrated the potential of this imaging technique in the imaging of iron deposition and micro-hemorrhages [10]–[12]. However, the breathing artifacts from long acquisition times have prevented the use of 3D SWI in the abdomen. 2D SWI (Work In Progress technique [WIP#608], Siemens Healthcare) is a new approach compared with 3D SWI, which is nearly immune to breathing artifacts because it takes advantage of breath-holds. This technique has been successfully applied to analysis of cirrhotic livers [13].

PH after liver cirrhosis is a direct cause of the development of esophageal varices (EV) and gastrointestinal bleeding. EV tends to increase in parallel with an increase in portal pressure. Bleeding from EV is the most important complication of cirrhosis. [14]. It is generally recommended that patients with liver cirrhosis undergo a screening endoscopy to look for the presence of EV; however, a large number of invasive endoscopic procedures turn out to be negative. Thus, there is a need for a non-invasive means to diagnose or predict the presence or absence of EV [15]. Several studies have evaluated possible non-invasive markers of EV in patients with PH and have found that platelet count, splenomegaly, portal vein diameter, and platelet count/spleen diameter ratio to be useful for this purpose [16]–[18].

The aims of this study were to assess the ability of 2D multislice breath-hold SWI to detect GGBs in the spleens of patients with PH caused by liver cirrhosis of different etiologies and to evaluate the potential role of GGB number as a non-invasive marker of PH and EV after a correlation analysis among the GGB number, the portal vein diameter, the splenic index (SI), the platelet count, and the platelet count/spleen diameter ratio.

## Materials and Methods

### Subjects

We analyzed 135 patients (103 males and 32 females, age range 21–74 years, mean age 47.6 years) with PH and 37 control individuals (23 males and 14 females, age range 21–60 years, mean age 44.3 years) who underwent consecutive MRI examinations from October 2010 to August 2011. A diagnosis of PH was based on clinical findings, a variety of biochemical markers, and imaging findings from an ultrasound, CT, and/or angiography [19]. The diagnostic criteria of PH were that patients had history of cirrhosis, spider naevi, esophageal varices, ascites, splenomegaly, and/or decrease in platelet count, decrease in platelet count/spleen diameter ratio, portal vein diameter ≥13 mm [19], [20]. The MR examination was performed within 1 to 7 days after PH was diagnosed. Inclusion criteria were patients with PH caused by liver cirrhosis of different etiologies. Patients were excluded from the study if their PH was caused by portal vein thrombosis or portal vein obstruction caused by hepatic hilar tumor. Patients were also excluded if their PH was associated with hematologic diseases, leukemia, lymphoma, benign or malignant tumors in the spleen.

All patients underwent a hematological and biochemical workup, including measurements of the total leukocyte count, platelet count, prothrombin time, and serum concentrations of bilirubin, albumin, alanine aminotransferase and aspartate aminotransferase.

The Medical Research Ethics Committee of the Third Military Medical University (Chongqing, China) reviewed and approved the present study. Written informed consent was obtained from each participant prior to the study.

### MRI protocol

All MRI scans were performed on a 3.0 T whole-body MRI system (MAGNETOM Trio, Siemens Healthcare, Erlangen, Germany) using a standard 12-channel matrix coil. The following MR-pulse sequences were used for all subjects: a transverse T1-weighted 2D gradient echo (GRE) (flip angle 70°, TR/TE 136/2.46 ms, pixel bandwidth, 280 Hz/pixel, 2 breath-holds), a transverse T2-weighted 2D fat-suppressed fast spin echo (flip angle 150°, TR/TE 3360/80 ms, pixel bandwidth, 260 Hz/pixel, respiratory-triggered), a coronal T2-weighted turbo spin echo (flip angle 150°, TR/TE 1000/90 ms, pixel bandwidth, 780 Hz/pixel, respiratory-triggered), a transverse T2*-weighted 2D GRE (flip angle 20°, TR/TE 150/10 ms, pixel bandwidth, 620 Hz/pixel, 3 breath-holds) and a transverse 2D SWI (flip angle 20°, TR/TE 150/10 ms, pixel bandwidth, 180 Hz/pixel, WIP#608). For all transverse sequences, the FOV was 380×280 mm, the matrix size was 380×280, the number of slices was 30, and the slice thickness was 5 mm with gaps of 1 mm. The protocol for SWI was similar to that previously described [13]. Three breath-holds were used, each lasting 16 seconds. The total acquisition time was no longer than 1 minute and 20 seconds, including break times between multi-breath-holds. SWI phase images were processed through a 32×32 highpass filter to remove background artifacts.

### Imaging analysis

All MRI images were reviewed in consensus by two senior radiologists, each with more than 10 years of experience in evaluating abdominal MR images, on a commercially available workstation (Syngo Multimodality Workplace, Siemens Healthcare). The gold standard for the confirmation of GGB in spleen of PH patients is pathological diagnosis. In our cohort, there are only two patients who received pathological evaluation of GGB in spleen, and we cannot confirm the GGB in this whole cohort. Based on the reasons that were put forward by previous study, the nodules, which were hypointense relative to background spleen parenchyma on T1-, T2-, T2*- weighted, and SWI images, were considered GGBs after excluding cross sections of blood vessels in the spleen [6]. The number of GGBs was determined from images obtained using the four MR sequences. For the convenience of calculation, a single representative slice, which by consensus contained the most GGBs, was chosen from the SWI dataset and was divided into 9 (3×3) equal-sized squares manually, then calculated the sum of GGB number from each square. The number of GGBs was counted on the corresponding T1-, T2-, and T2*- weighted slices that most closely matched the chosen SWI slice.

### Measurements of portal vein diameter, SI, and platelet count/spleen diameter ratio

**Figure 1:**
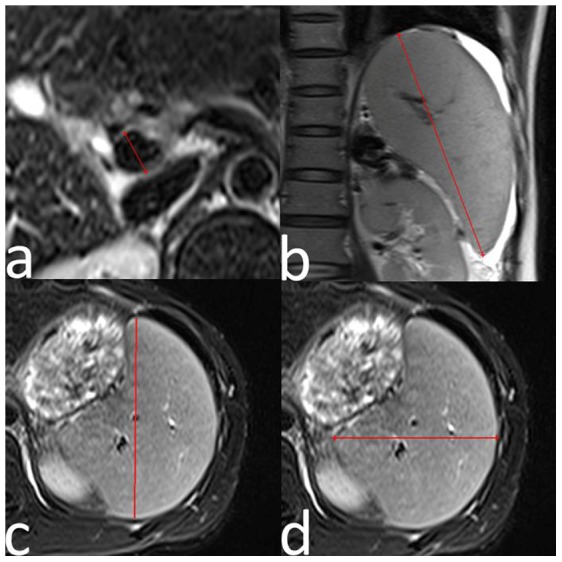
Measurements of portal vein diameter, SI, and platelet count/spleen diameter ratio. Measurement of portal vein diameter 3–4 slices above the junction of the superior mesenteric and splenic veins on transverse T2-weighted fat-suppressed images (a). Measurement of splenic craniocaudal (b), anteroposterior (c), and transverse (d) distance on coronal and axial T2-weighted images.

Portal vein diameter was measured 3–4 slices above the junction of the superior mesenteric vein and splenic vein on the transverse T2-weighted fat-suppressed images (Fig. 1a). SI  =  length×width×height of the spleen [21]. Spleen size was measured on the slice with the largest splenic craniocaudal, anteroposterior, and transverse distances on the coronal and axial T2-weighted images (Fig. 1b, c, and d). All measurements were performed on the MR workstation. The platelet count/spleen diameter ratio was calculated by dividing the platelet number by the maximum spleen bipolar diameter [16].

### Statistical analysis

Statistical analysis was performed using SPSS, version 17.0 software (SPSS, Chicago, Ill). The GGB detection rates on the T1-, T2-, T2*- weighted, and SWI images of all the 135 patients were evaluated with a chi-square test. The Mann-Whitney *U* test and Kruskal-Wallis test were used to compare the numbers of GGBs in the 19 patients with GGBs on all four imaging modalities. ANOVA was used to compare the portal vein diameter and SI among the 3 groups (37 controls, and the 84 and 51 PH patients were negative and positive, respectively, for GGBs on the SWI). Two-sample *t* tests were used to compare the platelet count and platelet count/spleen diameter ratio between the GGB-positive and GGB-negative groups (for GGBs on the SWI). The Spearman correlation analysis was used to assess the relationships among the GGBs number, portal vein diameters, SI, platelet count, and platelet count/spleen diameter ratio in the 51 SWI GGB-positive patients. A P-value < 0.05 was considered statistically significant. All measurements were expressed as the mean ± standard deviation.

## Results

### GGB detection rates and GGB numbers on T1-, T2-, T2*- weighted, and SWI images

**Figure 2:**
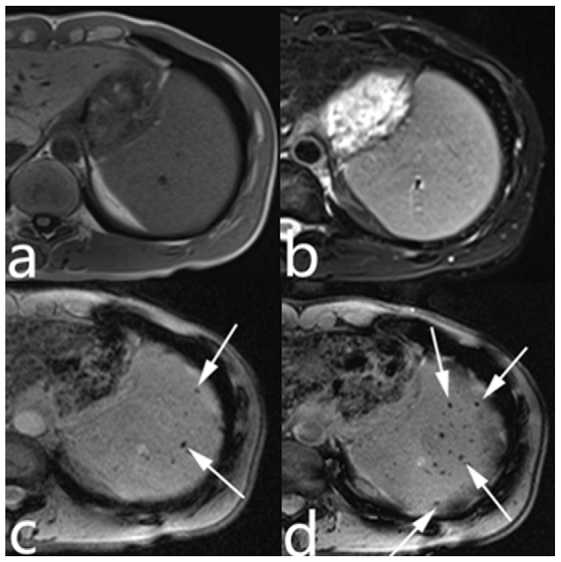
SWI detected significantly more patients with GGBs than did T1-, T2-, and T2*-weighted images. TIWI (a), T2WI (b), T2*WI (c), and SWI (d) images of a 36-year-old male patient with post-hepatitis cirrhosis. No GGBs were visible on T1WI(a) or T2WI (b); a few were visible on T2*WI (c) (arrows); and more were visible on SWI (d) (arrows).

**Figure 3:**
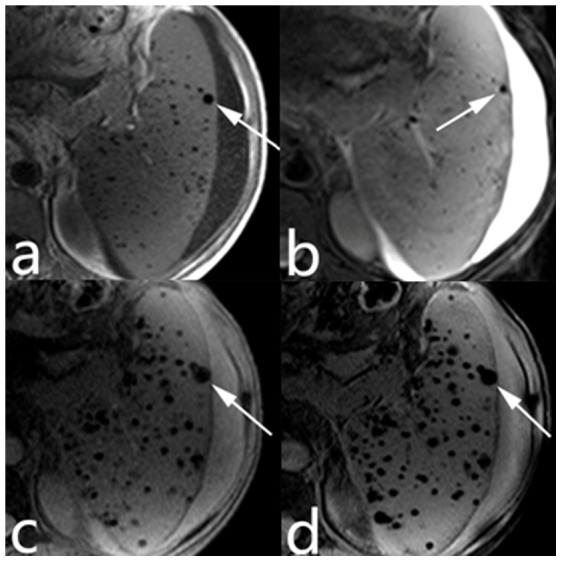
SWI detected more GGBs than did T1-, T2-, and T2*-weighted imaging. TIWI (a), T2WI (b), T2*WI (c), and SWI (d) images of a 45-year-old male patient with post-hepatitis cirrhosis, showing typical GGBs. Many GGBs smaller than 3 mm in diameter that were visualized on SWI and T2*WI were not visible on TIWI and T2WI. The diameters of the same GGBs were 5.7, 4.2, 8.8, and 9.2 mm on the TIWI, T2WI, T2*WI, and SWI images, respectively (arrow).

**Figure 4:**
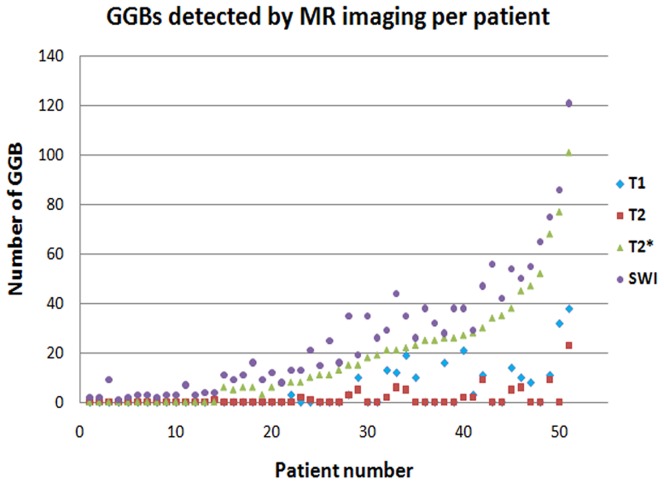
GGB numbers on T1WI, T2WI, T2*WI, and SWI images per patient. Number of GGBs per patient detected on a single representative slice of T1WI, T2WI, T2*WI, and SWI images.

GGBs in all sequences were visualized as low-signal nodules [3], [5], [6]. On the SWI images, the diameters of the GGBs were 1–12 mm. The identical nodules had larger diameters on the SWI than on T1- and T2-weighted images. The numbers of patient with GGBs in the spleen detected with the four MR sequences in the 135 PH patients, the number of GGBs on a single representative slice, the total number of GGBs <3 mm, and the number of GGBs >3 mm in the 19 patients with GGBs on all four imaging modalities are shown in Table 1. SWI detected significantly more patients with GGBs than did T1-, T2-, and T2*-weighted images (Fig. 2). The Kruskal-Wallis test showed that the GGB number were significantly different among the 4 sequences. The Mann-Whitney U test showed that SWI detected more GGBs than did T1-, T2-, and T2*-weighted imaging (Fig. 3, 4).

**Table 1:** The numbers of patient with GGBs in the spleen detected with four MR sequences in 135 PH patients, the numbers of GGBs and the total numbers of GGBs <3 mm in 19 patients with GGBs on all four imaging modalities.

	T1WI	T2WI	T2* WI	SWI	
Detection rate	22/135	19/135	38/135	51/135	*P*<0.05*
GGB Numbers	11.1±9.9	5.4±5.5	28.3±25.5	40.9±28.4	*P*<0.05**
Total numbers of GGBs <3 mm	58	24	166	335	*P*<0.05**
Total numbers of GGBs >3 mm	155	109	385	558	*P*<0.05**

* : chi-square test;

** : Kruskal-Wallis test.

### Comparison of portal vein diameter, SI, and platelet count among the controls, GGB-negative patients, and GGB-positive patients

The range of portal vein diameters, SIs, and platelet counts among the controls, GGB-negative, and GGB-positive patients (for GGBs on the SWI) are shown in Table 2. We observed a significant difference between the groups in these three parameters (*P* <0.05 each).

**Table 2:** Portal vein diameter, SI, and platelet count of controls, GGB-negative patients, GGB-positive patients (for GGBs on the SWI).

	Controls	GGB-negative	GGB-positive	
Portal vein diameter (mm)	10.79±1.21	13.29±1.95	16.13±2.61	*P*<0.05*
SI (cm^3^)	194.8±71.8	524.5±274.2	981.7±552.2	*P*<0.05*
PC(×mm^3^)	——	126455±41827	57372±23369	*P*<0.05**
PC/spleen diameter ratio	——	1076.1±421.2	396.7±184.2	*P*<0.05**

* : ANOVA test;

** : two-sample *t* test.

Results were represented as mean±standard deviation, SI: splenic index, PC: platelet count.

### Correlation of GGB numbers with portal vein diameter, SI, platelet count/spleen diameter ratio, and platelet count

**Figure 5:**
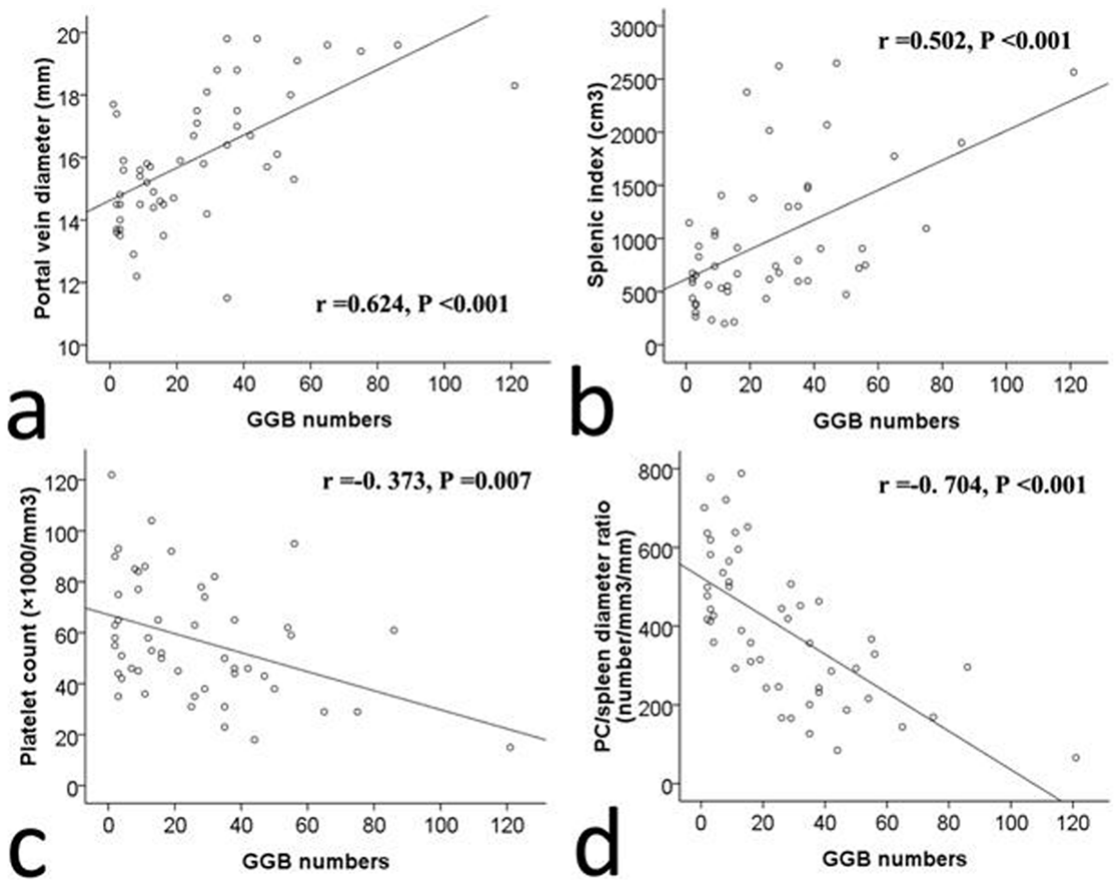
Correlation of GGB numbers with portal vein diameter, SI, platelet count/spleen diameter ratio, and platelet count. The GGB numbers counted on SWI images were significantly correlated with the portal vein diameter (a), SI (b), platelet count (PC) (c), and platelet count/spleen diameter ratio (d).

The GGB number counted on the SWI images were significantly correlated with portal vein diameter (r  = 0.624, *P*<0.001), SI (r  = 0.502, *P*<0.001), platelet count (r  = −0. 373, *P* = 0.007), and platelet count/spleen diameter ratio (r  = −0. 704, *P*<0.001). (Fig. 5). SWI shows better correlations with these clinical features of cirrhotic patients with PH, compared to T1-, T2- and T2*- weighted imaging (Table 3).

**Table 3:** The correlations among GGB numbers from T1WI, T2WI, T2*WI, SWI sequences and portal vein diameter, SI, platelet count, platelet count/spleen diameter ratio.

	Portal vein diameter	SI	Platelet count	Platelet count/spleen diameter ratio
T1WI	r = 0.327, P = 0.073	r = 0.427, P = 0.017	r = −0.198, P = 0.285	r = −0.459, P = 0.009
T2WI	r = 0.328, P = 0.102	r = 0.457, P = 0.019	r = −0.257, P = 0.184	r = −0.445, P = 0.020
T2*WI	r = 0.551, P <0.001	r = 0.368, P = 0.011	r = −0.228, P = 0.122	r = −0.512, P <0.001
SWI	r = 0.624, P <0.001	r = 0.502, P <0.001	r = −0. 373, P = 0.007	r = −0. 704,P <0.001

SI: spleen index.

## Discussion

PH of various etiologies can lead to splenomegaly and reticuloendothelial cell hyperplasia. A prolonged blood transit time and increased blood pressure in the enlarged spleen can cause micro-hemorrhages, which are phagocytosed by reticuloendothelial cells; these, in turn, produce hemosiderin adjacent to thickened collagen tissues and calcium deposits, eventually forming GGBs [22]. These lesions are also found in patients with hemolytic anemia, drepanocytic anemia, leukemia, lymphoma, and acquired hemochromatosis [22], [23].

Previous studies showed that MRI was superior to both ultrasonography and CT in identifying GGBs, resulting in detection rates of 9–12% [5], [8], [24]. Our results show that the detection rate of GGBs on SWI images was 37.8% (51/135), which was significantly higher than the rates from T1-, T2-, and T2*-weighted sequences. Several factors account for this improvement. First, compared with the T1-, T2-, and T2*-weighted sequences, SWI is more sensitive to paramagnetic substances because it uses phase information to enhance susceptibility effects in the images. The standard T2*-weighted sequence also has magnitude and phase data, which most of the time, the phase data is thrown away. The second factor is the “blooming effect”, by which a highly paramagnetic object less than a quarter of a voxel in area can have a dramatic appearance within a single voxel [25]; this effect enables more and larger GGBs to be visualized on SWI than on T1-, T2-, and T2*-weighted images. For this reason, the quantitative susceptibility imaging was performed in current study as a means of being less affected by blooming artifacts. The third factor is the use of the breath-holding 2D acquisition technique and the processing of data from each of the 12 acquisition channels individually; the technique is basically immune to breathing artifacts and the cusp artifacts resulting from the B_0_ inhomogeneity of 3T MR scanners [13].

Improved detection of GGB has possible clinical implications and avenues for future research. Progressive hepatic fibrosis results in architectural changes in cirrhotic liver and increases the portal vein pressure. EV bleeding is a serious complication of PH with significant morbidity and mortality [26]. Primary prophylaxis with nonselective beta-blockers and endoscopic band ligation may reduce the risk of variceal bleeding [27]. Therefore, it is generally recommended that patients with cirrhosis undergo endoscopic screening for EV at the time of diagnosis. Portal vein pressure can be measured directly during the performance of a transjugular intrahepatic portal systemic shunt (TIPS) for the treatment of complications of PH [28] or can be measured as the hepatic venous pressure gradient (HVPG), which is the difference between the occluded hepatic venous pressure and the free hepatic venous pressure [29]. A previous study showed that the risk of EV bleeding is significantly less if the HVPG falls to less than 12 mm Hg or by more than 20% [30]. As a result of the costly and invasive nature of TIPS, HVPG, and endoscopic screening, there is interest in developing a non-invasive predictor of the presence and development of PH and EV that would decrease the number of endoscopies performed.

Several studies have evaluated possible non-invasive markers of PH and EV in patients with cirrhosis and have found platelet count, splenomegaly, high portal vein diameter, and platelet count/spleen diameter ratio to be useful for this purpose [16], [17], [21], [31], [32]. A previous study indicated that the number of GGBs has been found to correlate positively with splenomegaly caused by PH [33]. Giannini et al. proposed using the platelet count/spleen diameter ratio as a non-invasive tool to predict the presence of EV, resulting in very high positive and negative predictive values [16]. Our results showed that the portal vein diameter, SI, platelet count, and platelet count/spleen diameter ratio differed significantly among control individuals and GGB-negative and GGB-positive patients. The number of GGBs correlated positively with the portal vein diameter and SI and correlated negatively with the platelet count and platelet count/spleen diameter ratio in the 51 patients positive for GGB on the SWI. This finding provides further evidence that GGB formation in the spleen is due to congestive splenomegaly caused by PH; the finding also indicates that GGB number may have a potential role in grading of PH and may be a non-invasive marker for improving the selection for endoscopic screening of patients at risk of EV.

However, GGBs are also found in patients with hemolytic anemia, drepanocytic anemia, leukemia, lymphoma, and acquired hemochromatosis, that should be taken into account when using GGB numbers to predict the presence of EV in PH patients associated with the above diseases.

There are several limitations to the current study. First, we did not measure the HVPG; therefore, without a correlation analysis between GGB number and HVPG, we could not directly assess the role of GGB number in grading PH. Second, there were only 9 GGB-positive patients who underwent endoscopic screening for EV, so we were unable to analyze the relationship between the GGB number and the degree of EV. Third, the numbers of GGBs were counted on single slices of SWI images, which may not fully indicate the presence of GGB in spleen.

It will be important in future research to analyze the relationship among GGB number, HVPG, and portal vein pressure in PH patients treated for complications with TIPS. It would be advantageous to develop the use of SWI for the spleen if a correlation analysis was performed between GGB number and degree of EV in future studies.

In conclusion, SWI provided more accurate information than T1-, T2-, and T2*-weighted images on GGBs in patients with PH caused by different etiologies. It also provided further evidence that the GGBs' formation in spleen is due to congestive splenomegaly caused by portal hypertension. GGB number may have a potential role in grading of PH and may be a non-invasive marker for improving the selection for endoscopic screening of PH patients at risk of EV.
